# The association between different blood group systems and susceptibility to COVID-19: a single center cross-sectional study from Saudi Arabia

**DOI:** 10.4314/ahs.v22i4.59

**Published:** 2022-12

**Authors:** Nora Y Hakami, Afnan J Al-Sulami, Wafaa A Alhazmi, Mamdouh M Sindi, Ohoud F Alotaibi, Maha A Badawi, Raed I Felimban, Thoraia M Shinawi, Talal Qadah

**Affiliations:** 1 Department of Medical Laboratory Technology, Faculty of Applied Medical Sciences, King Abdulaziz University, Jeddah, Saudi Arabia; 2 Blood Transfusion Services, King Abdulaziz University Hospital, Jeddah, Saudi Arabia; 3 Clinical Chemistry Laboratory, King Abdulaziz University Hospital, Jeddah, Saudi Arabia; 4 King Faisal Medical Complex, Taif, Saudi Arabia; 5 Department of Hematology, Faculty of Medicine, King Abdulaziz University, Jeddah, Saudi Arabia; 6 Hematology Research Unit, King Fahd Medical Research Center, King Abdulaziz University, Jeddah, Saudi Arabia; 7 Center of Innovation in Personalized Medicine, King Abdulaziz University, Jeddah, Saudi Arabia

**Keywords:** ABO Blood group, Rh(D), P1 antigen, COVID-19

## Abstract

**Background:**

Since the beginning of COVID-19 pandemic, many associated factors have been investigated to clarify the susceptibility and severity among the affected individuals. Biological markers can play an important role in identification of individual susceptibility to such pandemic. Growing evidence suggest the influence of different blood group systems on susceptibility to COVID-19 virus, with a particular blood type conferring selection advantage.

**Objectives:**

The study aimed to determine the association of ABO, Rhesus (D) and P1 blood groups with COVID-19 susceptibility in Taif city, Western Saudi Arabia.

**Methods:**

ABO, D and P1 blood antigens were determined in 104 blood samples of COVID-19 patients versus 100 control samples using either automated immunohematology analyser or test tube method. Statistical differences between patients and control samples were calculated based on p-value where results of ≤ 0.05 were considered significant.

**Results:**

O+ve blood group constituted the predominant type among the studied samples. Determination of P1 antigen showed significant association where Anti-P1 was positive in 76.9% of patients compared to 61.0% of controls with a P value of 0.01 conferring the susceptibility of P1+ve patients to COVID-19.

**Conclusion:**

Although our study showed no significant association between ABO and D, and susceptibility to COVID-19, there was a significant association between P1+ve and COVID-19. P1+ve participants were 2.131 times more associated with the risk of COVID-19 infection than those with Anti P1-ve. Thus, P1 antigen can be used as a biological marker for identification of individuals susceptibility to COVID-19. It is strongly advised that such individuals should consider extra protective measures. Further studies on other contributing factors should also be considered for more scientific clarity.

## Introduction

The emergence of rapidly spreading novel coronavirus SARS-CoV-2 infection, causing the new infectious disease known as COVID-19 has become a major health issue worldwide as the World Health Organization (WHO) has declared a pandemic status for this highly contagious disease^1^,^2^. The status has highlighted on the importance of finding rapid biological markers for predicting individual susceptibility to COVID-19 infection. Our knowledge about different blood groups' system can be utilized scientifically as predictive markers.

ABO and Rhesus (D) blood group antigens play an important role in transfusion safety, and disease exposures^3^. The most important blood group system in humans is the ABO blood group which comprises of 4 blood types, namely, A, AB, B, and O. The second most important blood group in humans is the Rh blood group, which has 49 antigens^4^. One of the most important is the D antigen and the Rh (D) status of an individual is usually indicated with a positive (+) or negative (−) suffix after the ABO type. It has been reported that there is a potential association between ABO phenotypes and an increased risk for disease susceptibility^3^,^5^. The frequencies of blood antigen groups (ABO and Rh) phenotypes are considerably varied among the populations^5^,^6^. This observation made the scientists to raise the question of their role in individual susceptibility to various diseases including infectious and non-infectious diseases. One of the first proven association of blood group polymorphism with disease sensitivity was between blood group O and peptic ulceration^5^. In addition, several reports have investigated the relationship between ABO blood groups and infectious diseases, particularly viral infections^7^–^11^. For example, the association between ABO blood group and the development of severe acute respiratory syndrome coronavirus (SARS-CoV1) infection was previously investigated in China suggesting that O blood group was considered as a protective factor against SARS-CoV1 infection compared to non O blood group^8^. A previous study has also reported blood groups association with the severity of dengue viral infection. It was noticed that blood group AB patients were more susceptible to have dengue hemorrhagic fever 9. Despite of the few numbers of diseases that were associated with Rh phenotypes compared to ABO, Rh factor is known to be essential in blood type compatibility and immune response^3^.

The association of SARS-CoV1 with ABO blood group directed towards the possibility of a similar susceptibility to the novel coronavirus SARS-CoV-2, which was explored by numerous researchers in the last 2 years. Several epidemiological and clinical studies provided growing evidence on the substantial role the ABO blood groups play in the immunopathogenesis of COVID-19. These include small observational studies to GWS (Genome wide association) which indicated the blood group O played a protective role, whereas blood group A conferred a higher risk COVID-19 susceptibility^12^–^16^.

Many other blood group systems have been listed by the International Society of Blood Transfusion involving over 379 antigens^17^. P blood group is considered one of the carbohydrate blood systems, having similar basic structure as ABO blood group system, known as ABO-like system. P1 is the most common phenotype having up to 90% of individual possessing P1 antigen on their red cells that can act as a receptor for several pathogens and toxins^18^. Consequently, it can be considered as a potential biological marker for predicting susceptibility to infectious diseases. With the several challenges posed by COVID-19 to the clinicians, the identification of potential biomarkers would provide crucial information that would aid in the timely diagnosis, confirmation of disease severity, identifying high risk individuals, predicting outcomes, patient care, etc.^19^. The identification of biological markers including ABO, D and P1 blood groups for predicting high risk individuals to COVID-19 through simple and rapid tests is important. It can provide a vital role in maintaining current cases under control. Furthermore, numerous recent reports have shown the association of ABO blood type with risk of COVID-19 illness^20^–^23^. Therefore, the main aim of this study was to identify the association of ABO, Rh and P1 blood groups with the COVID-19 infection to determine if these blood antigens can be used as biological markers for predicting the susceptibility of individuals to the COVID-19 infection.

## Methods

### Study design and Sample Collection

This is a cross sectional study that was conducted between September and November 2020. A total of 104 and 100 blood samples were retrieved from the database of patients with COVID-19 and healthy individuals respectively in King Faisal Medical Complex at Taif city, Western Saudi Arabia. There were no eligibility criteria, except being positive for COVID-19. The ethical approval was obtained from the directorate of health affairs - Taif with IRB number HAP-02-T-067.

### Inclusion and Exclusion Criteria

Adult healthy individuals and patients diagnosed with COVID-19 were approached by filling an electronic form containing purpose and the importance of the study as well as the type of samples to be taken. For the COVID-19 patients, inclusion criteria included confirmed infection with the virus while for the control group, no previous infection with the COVID-19 virus. Normal individuals and infected patients not consented were excluded from the study

### Phenotypic identification of ABO, D and P1 blood antigens

Determination of the RBCs antigens was performed using various serologic methods (i.e., antibodies against ABO, D and P1 specific antigens). The “phenotype” of any blood group refers to which antigens are detectable on the RBCs.

The recruited samples were analysed for ABO and D antigens using automated immunohematology analyser (Autovue blood group analyser, Switzerland). For P1 antigen detection, single antigen testing method was utilized using Bio-Rad DiClon® Anti-P1 blood group reagent and its identification was performed following test tube technique in accordance with manufacture's instruction for presence or absence of the P1 antigen (Hemagglutination). P1 phenotype was selected since it is the most common phenotype among others.

### Diagnosis of COVID-19

Real-Time Reverse Transcriptase Polymerase-Chain-Reaction method was utilized using Logix Smart™ Coronavirus Disease 2019 (COVID-19) Kit and performed on nasal and pharyngeal swab specimens from suspected patients. The process of confirmation included 3 stages: sample preparation, reverse transcription, and the polymerase chain reaction with real-time monitoring. All steps were followed as per company's instructions.

### Data analysis

All data were analysed using Microsoft excel and the statistical analysis for all data was performed using the IBM SPSS® software for Windows, version 23 (IBM SPSS, IBM Corp., Armonk, N.Y., USA). The results were presented as percentage number (%) and analyzed by the software IBM SPSS® Statistics. The Chi Square test was used to compare patients and control. Cross tabulation between groups and risk factor was performed followed by calculating the Odd's ratio and 95% confidence interval (CI). *P-value* of <0.05 were considered statistically significant.

## Results

### Distribution of ABO blood groups among study participants

**Figure 1:**
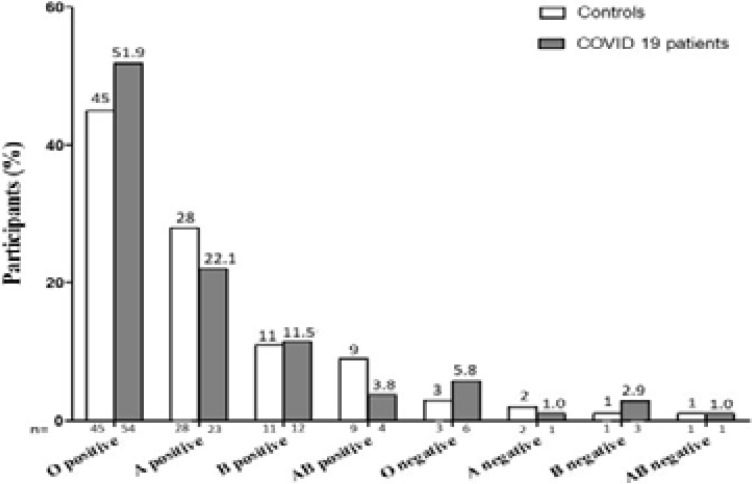
The distribution of Blood groups among the study participants. Values are expressed as number (%).

The blood groups in COVID-19 patients and control are shown in figure 1. The percentage of both positive and negative blood types O, A, B, and AB were 48%, 30%, 12%, and 10%, respectively in the Control group, while among the COVID-19 group the percentages were 57.7%, 23.1%, 14.4%, and 4.8%, respectively. In the control, the blood groups of participants were mainly composed of O positive (45.0%), then A positive (28.0%), B positive (11.0%), AB positive (9.0%), O negative (3.0%), A negative (2.0%) and lastly B negative (1.0%) and AB negative (1.0%). While, In the COVID-19 patients, the blood groups of participants were mostly O positive (51.9%), then A positive (22.1%), B positive (11.5%), O negative (5.8%), AB positive (3.8%), B negative (2.9%) and lastly A negative (1.0%) and AB negative (1.0%).

Overall, if we looked at the percentage of individuals in our study with different blood groups infected with COVID-19, then we found O+ve individuals (51.9%) to have the highest risk of infection than other blood groups.

### The association of ABO Rh and P1 antigens to the COVID-19 susceptibility

The Rh factor was positive in 89.4% of COVID-19 patients and in 93.0% of controls with insignificant difference between them (P =0.258). The odds ratio, though not significant (P =0.258) suggested that the odds of being infected with COVID-19 was 1.571 times more if the patient is Rh positive than being Rh negative (Table 1). The Rh factor was positive in 89.4% of COVID-19 patients and in 93.0% of controls with insignificant difference between them (P =0.258).

**Table (1):** Odd's ratio of Rhesus (Rh) factors in patients and control

Rh Factor	Control (n= 100)	COVID- 19 patients (n= 104)	P value	Odd's ratio	95% Confidence interval (CI)
**Rh negative**	7 (7.0%)	11 (10.6%)	0.258	1.571	0.584 – 4.230
**Rh positive**	93 (93.0%)	93 (89.4%)			

Values are expressed as number (%). Significance between patients and controls was calculated using Chi-Square test.

The Anti-P1 antigen was positive in 76.9% of COVID-19 patients and 61.0% of controls with significant difference between them (P =0.010). With a 95% CI (1.16–3.91), the odds ratio significantly (P =0.010) suggested that the odds of being infected with COVID-19 was 2.131 times more if the patient is positive for Anti-P1 antigen (Table 2).

**Table (2):** Odd's ratio of Anti-P1 factors in patients and control

Anti P1	Control (n= 100)	COVID- 19 patients (n= 104)	P value	Odd's ratio	95% Confidence interval (CI)
**Negative**	39 (39.0%)	24 (23.1%)	**0.010**	2.131	1.160 – 3.914
**Positive**	61 (61.0%)	80 (76.9%)			

Values are expressed as number (%). Significance between patients and controls was calculated using Chi-Square test.

Blood group AB was found in 4.8% and 10.0% of COVID-19 patients and controls respectively, with insignificant difference between them (P = 0.12). This indicates that individuals with blood group AB may have the least low risk of infection with COVID 19 than other individuals with other blood groups (Odd's ratio 0.455, 95% CI: 0.15–1.38) as shown in (Table 3). Similarly, blood group A was found in 23.1% and 30.0% of COVID-19 patients and controls respectively, with insignificant difference between them (P =0.168). Participants with blood group A had a lower risk of developing an infection with COVID-19 than individuals of blood group AB and others (Odd's ratio 0.700, 95% CI: 0.375–1.308). Blood group B was found in 14.4% and 12.0% of COVID-19 patients and controls respectively with insignificant difference between them (P = 0.38). It can be inferred that individuals with blood group B showed a higher risk of infection with COVID 19 than those with other blood groups (Odd's ratio 1.236, 95% CI: 0.547–2.790). Furthermore, we found that blood group O was found in 57.7% and 48.0% of COVID-19 patients and controls respectively, with insignificant difference between them (P =0.106). Interestingly, individuals with blood group O may have the highest risk of infection with COVID-19 among all other groups (Odd's ratio 1.477, 95% CI: 0.850–2.567) as shown in (Table 3).

**Table (3):** Odd's ratio of ABO blood groups in patients and control

ABO blood group	Control (n= 100)	COVID- 19 patients (n= 104)	P value	Odd's ratio	95% Confidence interval (CI)
**Group AB**					
**Negative**	90 (90.0%)	99 (95.2%)	0.124	0.455	0.150 - 1.380
**Positive**	10 (10.0%)	5 (4.8%)			
**Group A**					
**Negative**	70 (70.0%)	80 (76.9%)	0.168	0.700	0.375 - 1.308
**Positive**	30 (30.0%)	24 (23.1%)			
**Group B**					
**Negative**	88 (88.0%)	89 (85.6%)	0.381	1.236	0.547 - 2.790
**Positive**	12 (12.0%)	15 (14.4%)			
**Group O**					
**Negative**	52 (52.0%)	44 (42.3%)	0.106	1.477	0.850 - 2.567
**Positive**	48 (48.0%)	60 (57.7%)			

Values are expressed as number (%). Significance between patients and controls was calculated using Chi-Square test.

## Discussion

With the World Health Organization (WHO) declaring the novel coronavirus SARS-CoV-2 infection outbreak a global pandemic, there is an urgent need for identifying rapid biological markers for predicting individuals' susceptibility to COVID-19 infection. The human blood grouping system has been widely explored and used as a first line of diagnostic biomarker for various infectious and non-infectious diseases in clinical setup^7^–^11^,^24^.

This study aimed to determine if there is an association between ABO, Rhesus (D) and P1 blood group blood type with the susceptibility of COVID-19 in Taif city, Western Saudi Arabia. We analysed a total of 104 COVID-19 patients' blood samples and 100 healthy control blood samples from King Faisal Medical Complex at Taif city. We found that the major prevalent blood group phenotype among the study participants was the blood type group O contributing to 57.7% of COVID-19 patient samples and 48% for healthy controls. Similar other studies have rather identified blood group A to be significantly more frequent than O blood group among COVID-19 patients as compared to control^25^. In another study, blood group O had the lowest prevalence of disease positivity and blood type A had a lower frequency than blood types B and AB^14^.

Further, we looked at the association between Rhesus (D) blood group on the susceptibility to COVID-19 infection and identified 89.4% of COVID-19 patients being Rh positive as compared to 93.0% of control sample, but the Rh negative and positive status did not have significant predictive effects on the susceptibility to COVID-19 (P =0.258). The study by Goker et al^25^, showed similar non-significant results for their analysis on the effect of Rh type on the clinical outcomes of COVID-19 patients. Other similar study has reported that Rh+ types were associated with a high risk for COVID-19 infection (15) This same study has also shown that individuals with B blood type were considerably at high risk to develop a severe illness of COVID-19^15^.

To further elucidate the association of other clinical important blood groups with COVID-19 our study revealed that the presence of P1 antigen may influence the susceptibility risk of COVID-19 infection. This was one of the most significant and interesting finding from our study where we found significant association of positive Anti-P1 antigen (P =0.010), with COVID-19. We identified that 76.9% of COVID-19 patients were positive for Anti-P1 antigen as compared with 61.0% of controls. This indicated that being infected with COVID-19 was more likely if the patient is positive for Anti-P1 antigen. Our findings showed that Anti-P1 positive participants had 2.13 times increase risk of COVID-19 infection with 95% CI (1.160–3.914). P1 antigen is commonly expressed on RBC and can act as receptors for several pathogens and toxins^18^. Previous studies showed that P1 antigen may facilitate bacterial and viral adhesion causing agglutination of RBCs^26^–^28^. In a previous attempt to characterize the adhesion specificity of Streptococcus suis, it was found that the bacteria agglutinate best among P blood group erythrocytes indicating a preference binding specificity 28. Similarly, *Pseudomonas aeruginosa* lectin I displayed high affinity to Pk and P1 antigens causing chronic lung diseases with fatal consequences^29^. The association between viral infections and P blood group system has been studied before and Brown et al was the first to report a preference binding of parvovirus B19 to RBCs with P antigen^30^. Our work showed similar finding in term of positivity to P1 blood group and this P1+ve association with COVID-19 susceptibility appears to be a novel finding, at least in the Saudi Arabia region and warrants further investigation. We further investigated the association of four different ABO blood groups in COVID-19 patients and control. In this cross-sectional study for Taif city, Western Saudi Arabia we failed to find any significant association between ABO blood type and COVID-19 infection susceptibility. Though several publications have indicated a role of ABO blood typing in COVID-19 acquisition and severity, this was not the case for us and similar other studies^3^,^8^,^31^. A recent study performed by Zhao et al has examined the relationship between the ABO blood group and the COVID-19 risk in Wuhan and Shenzhen, China. It was found that individuals with blood group A showed a higher susceptibility risk for COVID-19 compared to non-A blood groups. On the other hand, individuals with blood group O had a lower risk for the infection compared to non-O blood groups^20^. The results of Zhao et al agreed with the meta-analysis study conducted in Italy and Spain, which also reported a higher risk of COVID-19 among blood group A with a reduced risk of infection among the blood group O individuals^21^. In contrast, our data showed that participants of blood group AB, A and B were at lower risk of infection with COVID-19 compared to blood group O which forms the highest susceptibility risk for COVID-19 infection. The discrepancy in the susceptibility of individuals to COVID-19 with regard to blood groups between the previous mentioned studies remains unclear and inconsistent. However, it can be inferred that susceptibility to COVID-19 infection may depend on ethnic and geographical location where the commonest blood group type in a specific region showed a predominance of susceptibility to COVID-19 infection. For example, the study of Zhao *et al* 20 where blood group A showed a higher susceptibility risk for COVID-19 in China is in consistent with a former study that investigated the ABO blood groups distribution among nine ethnic groups in China where blood group type A comprised the major prevalent type followed by B, O and AB blood groups^32^ and thus blood group A individuals are more susceptible to COVID-19. Another recent multi-institutional study performed in the state of Massachusetts, the United States of America supports our notion. It has been found that among the 1289 patients who tested positive, 440 (34.2%) were blood type A, 201 (15.6%) were blood type B, 61 (4.7%) were blood type AB, and 587 (45.5%) were blood type O. Although the study concluded that blood type O was less likely to test positive for COVID-19, the distribution of infected patients showed high percentage of blood group O 15. This agrees with the prevalence of ABO blood group types in US population where blood group O comprises the most common type^6^. In addition, limitation factors of such studies should be carefully considered when interpreting the association of ABO blood groups with susceptibility to COVID-19. This was evidenced by a retrospective cohort analysis on Danish individuals who tested positive for COVID-19 claiming a reduced prevalence of COVID-19 in ABO blood group O. The study has mentioned several limitations including availability of ABO blood group information that have been determined for only 62% of all tested individuals and more importantly blood group distributions varied among ethnic subgroups with different susceptibility for infection^23^. Another study performed in Peshawar region, Pakistan found a significant association between blood types B and AB and susceptibility to COVID-19 where there was no association between blood groups A and O with COVID-19. The study concluded that this variation may be related and relayed on genetic clustering of susceptible loci that may vary among different ethnicities^21^,^33^. It is important to mention the limitation of our study which includes the number of samples obtained from a single center. This is not unusual since other studies performed on the same topic from other countries have similar limitation^15^,^33^.

## Conclusion

The current study indicates that P1 antigens may have a potential role on the COVID-19 disease susceptibility; however, the exact mechanism by which these molecules confer sensitivity or protection still requires further investigations. Therefore, it is highly recommended to carry out further analysis to elucidate the underlying mechanisms. Also, given the lack of association between the ABO subtype and Rh blood group indicated by us and some other groups, the ABO blood typing should not currently be considered as a prognostic biomarker in those who acquire the disease. Prospective larger multicentre studies are required in order to elucidate further the role of ABO blood typing in COVID-19 susceptibility.

## References

[1] Lai CC, Shih TP, Ko WC, Tang HJ, Hsueh PR (2020). Severe acute respiratory syndrome coronavirus 2 (SARS-CoV-2) and coronavirus disease-2019 (COVID-19): The epidemic and the challenges. Int J Antimicrob Agents.

[2] Cheng ZJ, Shan J (2020). 2019 Novel coronavirus: where we are and what we know. Infection.

[3] Liumbruno GM, Franchini M (2013). Beyond immunohaematology: the role of the ABO blood group in human diseases. Blood Transfus.

[4] Dean L (2005). The Rh Blood groups. Blood Groups and Red Cell Antigens [Internet].

[5] Anstee DJ (2010). The relationship between blood groups and disease. Blood.

[6] Garratty G, Glynn SA, McEntire R (2004). Retrovirus Epidemiology Donor S. ABO and Rh(D) phenotype frequencies of different racial/ethnic groups in the United States. Transfusion.

[7] Lindesmith L, Moe C, Marionneau S, Ruvoen N, Jiang X, Lindblad L (2003). Human susceptibility and resistance to Norwalk virus infection. Nat Med.

[8] Cheng Y, Cheng G, Chui CH, Lau FY, Chan PK, Ng MH (2005). ABO blood group and susceptibility to severe acute respiratory syndrome. JAMA.

[9] Kalayanarooj S, Gibbons RV, Vaughn D, Green S, Nisalak A, Jarman RG (2007). Blood group AB is associated with increased risk for severe dengue disease in secondary infections. J Infect Dis.

[10] Wang DS, Chen DL, Ren C, Wang ZQ, Qiu MZ, Luo HY (2012). ABO blood group, hepatitis B viral infection and risk of pancreatic cancer. Int J Cancer.

[11] Kumar NC, Nadimpalli M, Vardhan VR, Gopal SD (2010). Association of ABO blood groups with Chikungunya virus. Virol J.

[12] Barnkob MB, Pottegård A, Støvring H, Haunstrup TM, Homburg K, Larsen R (2020). Reduced prevalence of SARS-CoV-2 infection in ABO blood group O. Blood Adv.

[13] Hoiland RL, Fergusson NA, Mitra AR, Griesdale DEG, Devine DV, Stukas S (2020). The association of ABO blood group with indices of disease severity and multiorgan dysfunction in COVID-19. Blood Adv.

[14] Zhao J, Yang Y, Huang H, Li D, Gu D, Lu X (2021). Relationship Between the ABO Blood Group and the Coronavirus Disease 2019 (COVID-19) Susceptibility. Clin Infect Dis.

[15] Latz CA, DeCarlo C, Boitano L, Png CYM, Patell R, Conrad MF (2020). Blood type and outcomes in patients with COVID-19. Ann Hematol.

[16] Li J, Wang X, Chen J, Cai Y, Deng A, Yang M (2020). Association between ABO blood groups and risk of SARS-CoV-2 pneumonia. Br J Haematol.

[17] Patnaik SK, Helmberg W, Blumenfeld OO (2014). BGMUT Database of Allelic Variants of Genes Encoding Human Blood Group Antigens. Transfus Med Hemother.

[18] Hellberg A, Westman JS, Thuresson B, Olsson ML (2013). P1PK: the blood group system that changed its name and expanded. Immunohematology.

[19] Samprathi M, Jayashree M (2020). Biomarkers in COVID-19: An Up-To-Date Review. Front Pediatr.

[20] Zhao J, Yang Y, Huang H, Li D, Gu D, Lu X (2020). Relationship between the ABO Blood Group and the COVID-19 Susceptibility. Clin Infect Dis.

[21] Barriteau CM, Bochey P, Lindholm PF, Hartman K, Sumugod R, Ramsey G (2020). Blood transfusion utilization in hospitalized COVID-19 patients. Transfusion.

[22] Zietz M, Zucker J, Tatonetti NP (2020). Associations between blood type and COVID-19 infection, intubation, and death. Nat Commun.

[23] Barnkob MB, Pottegard A, Stovring H, Haunstrup TM, Homburg K, Larsen R (2020). Reduced prevalence of SARS-CoV-2 infection in ABO blood group O. Blood Adv.

[24] Groot HE, Villegas Sierra LE, Said MA, Lipsic E, Karper JC, van der Harst P (2020). Genetically Determined ABO Blood Group and its Associations with Health and Disease. Arterioscler Thromb Vasc Biol.

[25] Göker H, Aladağ Karakulak E, Demiroğlu H, Ayaz Ceylan ÇM, Büyükaşik Y, Inkaya AÇ (2020). The effects of blood group types on the risk of COVID-19 infection and its clinical outcome. Turkish journal of medical sciences.

[26] Moulds JM, Moulds JJ (2000). Blood group associations with parasites, bacteria, and viruses. Transfus Med Rev.

[27] Ziegler T, Jacobsohn N, Fünfstück R (2004). Correlation between blood group phenotype and virulence properties of Escherichia coli in patients with chronic urinary tract infection. Int J Antimicrob Agents.

[28] Haataja S, Tikkanen K, Liukkonen J, Francois-Gerard C, Finne J (1993). Characterization of a novel bacterial adhesion specificity of Streptococcus suis recognizing blood group P receptor oligosaccharides. J Biol Chem.

[29] Blanchard B, Nurisso A, Hollville E, Tetaud C, Wiels J, Pokorna M (2008). Structural basis of the preferential binding for globo-series glycosphingolipids displayed by Pseudomonas aeruginosa lectin I. J Mol Biol.

[30] Brown KE, Anderson SM, Young NS (1993). Erythrocyte P antigen: cellular receptor for B19 parvovirus. Science.

[31] Harris JB, LaRocque RC (2016). Cholera and ABO Blood Group: Understanding an Ancient Association. Am J Trop Med Hyg.

[32] Liu J, Zhang S, Wang Q, Shen H, Zhang Y, Liu M (2017). Frequencies and ethnic distribution of ABO and RhD blood groups in China: a population-based cross-sectional study. BMJ Open.

[33] Rahim F, Amin S, Bahadur S, Noor M, Mahmood A, Gul H (2021). ABO / Rh-D Blood types and susceptibility to Corona Virus Disease-19 in Peshawar, Pakistan. Pakistan Journal of Medical Sciences.

